# Decoding the Promise and Challenges of miRNA-Based Cancer Therapies: An Essential Update on miR-21, miR-34, and miR-155

**DOI:** 10.7150/ijms.102123

**Published:** 2024-10-28

**Authors:** Hongbo Qian, Mazaher Maghsoudloo, Parham Jabbarzadeh Kaboli, Ali Babaeizad, Yulan Cui, Junjiang Fu, Qingjing Wang, Saber Imani

**Affiliations:** 1 Shulan International Medical College, Zhejiang Shuren University, Hangzhou, Zhejiang, China.; 2 Key Laboratory of Epigenetics and Oncology, The Research Center for Preclinical Medicine, Southwest Medical University, Luzhou 646000, Sichuan, China.; 3 Department of Biochemistry, Faculty of Medicine, Medical University of Warsaw, Warsaw 02-091, Poland.; 4 Faculty of Medicine, Semnan University of Medical Sciences, Semnan, Iran.

**Keywords:** miRNA therapy, miR-21, miR-34, miR-155, cancer treatment.

## Abstract

MicroRNAs (miRNAs)-based therapies hold great promise for cancer treatment, challenges such as expression variability, off-target effects, and limited clinical effectiveness have led to the withdrawal of many clinical trials. This review investigates the setbacks in miRNA-based therapies by examining miR-21, miR-34, and miR-155, highlighting their functional complexity, off-target effects, and the challenges in delivering these therapies effectively. Moreover, It highlights recent advances in delivery methods, combination therapies, and personalized treatment approaches to overcome these challenges. This review highlights the intricate molecular networks involving miRNAs, particularly their interactions with other non-coding RNAs, such as long non-coding RNAs (lncRNAs) and circular RNAs (circRNAs), emphasizing the pivotal role of miRNAs in cancer biology and therapeutic strategies. By addressing these hurdles, this review aims to steer future research toward harnessing the potential of miRNA therapies to target cancer pathways effectively, enhance anti-tumor responses, and ultimately improve patient outcomes in precision cancer therapy.

## Introduction

MicroRNAs (miRNAs) are key regulatory molecules that modulate gene expression at the post-transcriptional level 1, 2. In cancer biology, miRNA dysregulation is common and significantly contributes to tumor development, metastasis, and resistance to therapy 3. In recent years, numerous clinical trials aimed at evaluating the safety and efficacy of miRNA-based therapies. Despite early promising results, many clinical trials for miRNA-cancer therapies have been faced with unexpected complications, leading to the withdrawal of these treatments from clinical testing 4. The withdrawal of miRNA-cancer therapies from clinical trials can be attributed to several significant challenges, including the complexities of miRNA biology, unexpected off-target effects, inadequate drug delivery systems, and difficulties with target specificity 4. Furthermore, challenges with effective combination therapy have also limited the successful translation of these therapies into clinical practice 5, 6.

Among the vast array of miRNAs, miR-21, miR-34, and miR-155 have garnered significant attention due to their dysregulation across various cancer types, including gastric cancer, bladder cancer, triple-negative breast cancer (TNBC), cervical cancer, glioblastoma, glioma, acute myeloid leukemia (AML), testicular cancer, lung cancer, oral squamous cell carcinoma (OSCC), esophageal squamous cell carcinoma (ESCC), sinonasal cancers (SNCs), and colorectal cancer (CRC) 7-14. These miRNAs exhibit diverse functions, including regulating cell proliferation, apoptosis, migration, invasion, and tumor-microenvironment interactions. They participate in complex molecular networks, interacting with other non-coding RNAs, such as long non-coding RNAs (lncRNAs) and circular RNAs (circRNAs), to exert their regulatory effects on gene expression 13, 15-17. Understanding the mechanistic underpinnings of these miRNAs and their interactions with other molecular players is crucial for elucidating their roles in cancer pathogenesis and identifying potential therapeutic targets.

In this study, we aim to provide a comprehensive overview of the roles of miR-21, miR-34, and miR-155 in different types of cancer, focusing on their potential as diagnostic and prognostic biomarkers, as well as their involvement in cancer progression, metastasis, and drug resistance. We examine the molecular mechanisms driving their role in tumor development and the intricate networks they influence. Furthermore, we will explore the therapeutic potential of targeting these miRNAs and their associated pathways, highlighting both the opportunities and challenges in developing miRNA-based therapies for cancer treatment.

## A Crucial Update for miRNAs: miR-21, miR-34 and miR-155

Understanding how these miRNAs interact with their target genes and other non-coding RNAs will lead to more precise and effective cancer treatments 18. The diverse functions of miR-21, miR-34, and miR-155 in various cancer types underscore their significance as central regulators of cancer progression and treatment response. These miRNAs exhibit a wide range of functions, including the modulation of cell proliferation, apoptosis, migration, invasion, and tumor-microenvironment interactions 4. Furthermore, the complex molecular networks involving these miRNAs, including their interactions with other non-coding RNAs such as lncRNAs and circRNAs, have been elucidated in various cancer types 16, 17, 19. These interactions provide a deeper understanding of cancer pathogenesis mechanisms and offer new avenues for therapeutic intervention.

### Lung Cancer

Lung cancer is the leading cause of cancer-related deaths worldwide, and the identification of molecular mechanisms driving its progression is crucial for the development of targeted therapies. In lung adenocarcinoma (LUAD), the miR-21-5p/Ras Homolog Family Member B (RhoB) axis plays a crucial role in regulating epithelial-mesenchymal transition (EMT). Studies demonstrated that miR-21-5p knockdown inhibits migration, invasion, cisplatin resistance, and sphere formation in A549 cells. These effects were accompanied by the upregulation of E-cadherin and downregulation of Slug, both markers associated with EMT. Conversely, RhoB silencing restored EMT characteristics, indicating that miR-21-5p promotes EMT through downregulating RhoB 20. These findings underscore the role of miR-21-5p in lung cancer progression and its potential as a therapeutic target. In studies on head and neck squamous cell carcinoma (HNSCC), miR-21-5p was significantly overexpressed in HNSCC cells and demonstrated strong predictive ability. Two predictive genes, Alcohol Dehydrogenase 7 (ADH7) and Retinol Dehydrogenase 12 (RDH12), were significantly negatively correlated with miR-21-5p when downregulated in HNSCC. This suggests that miR-21-5p may target ADH7 and RDH12, contributing to the regulation of retinol metabolism 21. Developing strategies to inhibit miR-21-5p or restore RhoB expression could offer new approaches for managing LUAD, particularly regarding metastasis and drug resistance 22.

### OSCC

OSCC accounts for the majority of oral malignant tumors and is often diagnosed at advanced stages, leading to poor prognosis. A study focusing on miR-24, miR-200, and miR-34 expression changes in saliva samples from OSCC patients has provided promising insights into their potential as non-invasive biomarkers. The research, involving 30 OSCC patients and 30 healthy controls, utilized Real-Time PCR to measure miRNA levels. The results indicated a significant decrease in miR-34 expression in OSCC patients compared to healthy individuals 23. Given the ease and non-invasive nature of saliva sample collection, miR-34 presents itself as a viable biomarker for the early detection of OSCC, which can significantly improve patient outcomes by facilitating earlier and more effective interventions. Further research is needed to validate these findings in larger patient cohorts and to establish standardized protocols for miRNA detection in saliva 23. In addition to its potential as a diagnostic biomarker, miR-21 has been shown to play a crucial role in the progression from oral epithelial dysplasia (OED) to OSCC. A study analyzing saliva, blood, and tissue samples from patients diagnosed with OSCC, OED, and healthy controls demonstrated significantly higher miR-21 levels in OED and OSCC patients than in healthy individuals 24. Moreover, a negative correlation between miR-21 and Programmed Cell Death 4 (PDCD4) expression was observed, suggesting that miR-21 may promote tumor progression by downregulating PDCD4 25, 26. This research indicates that miR-21 could serve as a valuable biomarker for early detection and monitoring of OSCC, providing a non-invasive method for assessing disease progression. Targeting the miR-21/PDCD4 axis is also a therapeutic strategy for preventing the progression of OED to OSCC 7.

### ESCC

ESCC is a highly aggressive malignancy with a poor prognosis, and the identification of molecular mechanisms driving its progression is crucial for improving patient outcomes. In ESCC, miR-155 has been implicated in tumor progression by interacting with circRNAs 8. Specifically, circ_0000592 has been identified as a key regulator in ESCC by modulating miR-155-5p activity. Higher expression levels of circ_0000592 in ESCC are associated with poor prognosis, including reduced survival time and increased metastasis. Mechanistic studies have shown that circ_0000592 interacts with miR-155-5p to counteract the inhibitory effects on the target gene Frizzled 5 (FZD5). This interaction promotes ESCC cell viability, migration, and invasion, highlighting the potential of circ_0000592 as a therapeutic target. Downregulation of circ_0000592 has been shown to suppress ESCC cell activity and reduce tumor growth *in vivo*, suggesting that targeting the circ_0000592/miR-155-5p axis could be a novel therapeutic strategy for ESCC. These findings underscore the importance of understanding the molecular interactions and pathways involving miR-155 in ESCC pathogenesis and the potential for developing targeted therapies based on this knowledge 8. The diagnostic and prognostic potential of miR-155-5p and miR-1246 in OSCC has also been explored, shedding light on their roles in chemotherapy resistance. Differential expression patterns of miR-155 and downstream targets among oral cancer cell lines with varying levels of chemotherapy resistance suggest a potential link between miR-155 dysregulation and treatment response. These findings highlight the need for further research to elucidate the molecular mechanisms underlying miR-155-mediated chemotherapy resistance in OSCC and to develop strategies for overcoming this resistance 22.

### SNCs

SNCs are a heterogeneous group of malignancies with varying histological features and clinical behaviors, and the identification of molecular drivers and biomarkers is crucial for improving patient outcomes. The miR-34/miR-449 cluster has been identified as critical in the pathogenesis of SNCs through next-generation sequencing-based miRNome analysis 9. An innovative study employed Argonaute-2: mRNA immunoprecipitation followed by high-throughput sequencing to explore the regulatory role of miR-34/miR-449 in SNCs. This approach revealed that miR-34 and miR-449 target genes involved in RNA-DNA metabolic, transcript, and epigenetic processes, specifically highlighting serine/threonine kinase 3, chromosome 9 open reading frame 78, and striated muscle preferentially expressed protein 3 as direct targets 9. The deregulation of these targets is predictive of tumor progression, suggesting that miR-34/miR-449 could serve as valuable prognostic biomarkers and potential therapeutic targets in SNCs. This finding opens new avenues for personalized medicine in treating SNCs, emphasizing further research to translate these biomarkers into clinical practice and develop targeted therapies based on the miR-34/miR-449 regulatory network 9.

### CRC

CRC is a leading cause of cancer-related deaths worldwide, and the identification of molecular mechanisms driving its progression is crucial for improving patient outcomes. Extensive research in CRC has explored the relationship between miR-34 and the p53 tumor suppressor pathway. A study utilizing a CRISPR/Cas9 approach to inactivate miR-34a and miR-34b/c in the HCT116 CRC cell line revealed critical insights into their complementary roles in regulating EMT and autophagy, processes crucial for tumor growth and metastasis 27. The concurrent deletion of miR-34a and miR-34b/c led to a significant reduction in the suppression of cell proliferation following p53 activation. Additionally, these cells exhibited enhanced migration, invasion, EMT, and reduced sensitivity to the chemotherapeutic agent 5-FU. Notably, the combined inactivation of miR-34a/b/c was associated with an EMT signature, impaired gene repression by the p53-DREAM (p53-DR1, E2F, AP-2, MIZ-1) pathway, and increased autophagy, contributing to chemoresistance. These findings suggest that miR-34 could be targeted to enhance the efficacy of CRC therapies, particularly in overcoming resistance to chemotherapeutics 27.

Further research in CRC has highlighted the negative correlation between miR-34 and the phosphatidylinositol 3-kinase (PI3K)/protein kinase B (AKT)/mechanistic target of rapamycin (mTOR) signaling pathway, a critical axis for tumor growth and metastasis 28. Immunohistochemical analysis of colorectal adenocarcinoma samples revealed elevated p-PI3K, p-AKT, and mTOR proteins in tumor tissues compared to normal mucosa. Moreover, the expression of miR-34a and miR-34b was significantly lower in tumor tissues, and their levels were inversely correlated with the expression of the PI3K/AKT/mTOR pathway proteins. This indicates that miR-34 may inhibit tumor progression by downregulating this signaling pathway, thereby representing a potential therapeutic target for CRC 28.

In addition to its therapeutic potential, miR-21 has also been investigated as a non-invasive biomarker for early CRC detection. A study exploring the possibility of plasma miRNAs as biomarkers for CRC screening revealed a significant increase in miR-21 expression in CRC patients compared to healthy controls. This increase was correlated with age and tumor location, with notable differences observed between proximal and distal colon sites. Additionally, the study reported a significant downregulation of miR-145 in CRC patients' plasma. These findings suggest that assessing miR-21 and miR-145 plasma levels could be a promising non-invasive screening tool for early CRC detection, potentially reducing screening costs and improving patient outcomes 29. Recent studies have also shed light on the role of miR-21 in CRC progression through its interaction with lncRNAs, such as FAM30A. FAM30A is significantly downregulated in CRC tissues, and its expression is inversely correlated with tumor severity and patient prognosis. FAM30A has been shown to regulate miR-21-3p negatively, inhibiting CRC cell proliferation and metastasis. The overexpression of FAM30A leads to a notable suppression of these malignant behaviors, suggesting that FAM30A and miR-21-3p interaction is pivotal in CRC progression. Consequently, FAM30A is a potential prognostic indicator and therapeutic target in CRC 30. Furthermore, the impact of perioperative administration of Bifidobacterium triplex viable capsules on the serum levels of circulating miR-21-5p in CRC patients has been investigated. The findings revealed that radical surgery significantly reduces the levels of serum miR-21-5p, and the use of Bifidobacterium triplex capsules further assists in quicker perioperative recovery, likely through the modulation of miR-21-5p expression. This underscores the therapeutic potential of targeting miR-21-5p to enhance surgical outcomes and postoperative recovery in CRC patients 31.

### Gastric Cancer

Gastric cancer is a highly aggressive malignancy with limited treatment options, particularly for advanced-stage patients. Recent research has shed light on the role of circRNAs in gastric cancer progression 32. circWNK1 has emerged as a significant player, functioning as a tumor suppressor by sequestering miR-21-3p. This interaction inhibits the transforming growth factor-beta (TGF-β) signaling pathway, which is known to be activated in many cancers, including gastric cancer 33, 34. The downregulation of circWNK1 leads to reduced SMAD family member 7 (SMAD7) expression and subsequent activation of the TGF-β pathway, promoting gastric cancer cell proliferation, migration, invasion, and EMT. These findings highlight the potential of circWNK1 as a biomarker for diagnosis and treatment in gastric cancer, emphasizing the importance of the circWNK1/miR-21-3p/SMAD7 axis in regulating tumor progression 32.

### Bladder Cancer

Bladder cancer is one of the most prevalent malignant tumors globally, with a higher incidence in males. Recent research has identified a novel circRNA, circPGM5, crucial in bladder cancer progression. Derived from the PGM5 gene, circPGM5 is significantly under-expressed in bladder cancer tissues 35. Functional assays have revealed that circPGM5 inhibits bladder cancer cell proliferation, migration, and invasion by sponging miR-21-5p. This interaction upregulates mitogen-activated protein kinase 10 (MAPK10) expression, which subsequently affects the phosphorylation of the tumor suppressor forkhead box O3 (Foxo3a). The circPGM5/miR-21-5p/MAPK10/Foxo3a axis underscores the tumor-suppressing role of circPGM5 and highlights the oncogenic nature of miR-21-5p in bladder cancer. These findings provide new insights into the molecular mechanisms underlying bladder cancer progression and suggest potential therapeutic targets for intervention 35.

### TNBC

TNBC is a particularly aggressive subtype of breast cancer (BC) characterized by the absence of estrogen receptor, progesterone receptor, and HER2 amplification. TNBC patients face limited treatment options and poor prognosis, emphasizing the need for novel therapeutic strategies 14. Research has demonstrated that miR-21 plays a crucial role in TNBC by influencing macrophage polarization through exosome-mediated communication. The depletion of Ras-related protein Rab-5A (RAB5A) in TNBC cells results in a significant reduction in exosome secretion and impairs the polarization of macrophages toward an M2 phenotype, which is associated with tumor progression. miR-21, a pivotal component in these exosomes, enhances M2 polarization, promoting tumor growth and metastasis 36. The knockdown of RAB5A leads to reduced tumor formation and impaired recruitment of tumor-associated macrophages *in vivo*. These findings suggest that targeting the miR-21/RAB5A axis could disrupt the tumor-microenvironment interactions, offering new therapeutic strategies for TNBC. Understanding the complex interplay between cancer cells and the tumor microenvironment (TME) is crucial for developing effective treatments for this challenging BC subtype 14.

### Cervical Cancer

Cervical cancer is a major global health concern, with human papillomavirus (HPV) infection being a key risk factor. In cervical cancer, miR-21 regulates the tumor suppressor gene Reversion Inducing Cysteine-Rich Protein with Kazal Motifs (RECK) 31. Studies have shown an inverse correlation between miR-21 expression and RECK mRNA and protein levels. Silencing miR-21 using siRNAs results in increased RECK expression, inhibiting cell proliferation and migration. This mechanistic insight highlights miR-21's role in promoting cervical cancer progression by downregulating RECK. Consequently, both miR-21 and RECK present themselves as potential targets for gene therapy aimed at curbing cervical cancer growth and metastasis. Developing targeted therapies that modulate the miR-21/RECK axis could provide new avenues for the treatment of cervical cancer, complementing existing HPV vaccination and screening strategies 31.

### Testicular Cancer

Testicular cancer is the most common solid malignancy in young men, and early detection is crucial for successful treatment. MiR-21 has been identified as a promising serum biomarker for testicular cancer in a study that analyzed a set of nine miRNAs. Along with miR-29a and miR-106b, miR-21 demonstrated a sensitivity exceeding 93%, surpassing traditional serum tumor markers. The robust sensitivity and specificity of miR-21 make it a strong candidate for early testicular cancer detection and monitoring. This finding underscores the potential of miRNAs as reliable serum biomarkers, facilitating improved clinical decision-making in testicular cancer management. Further validation of these results in larger cohorts and prospective studies could lead to the development of a non-invasive, miRNA-based screening tool for testicular cancer 37.

### Glioblastoma

Glioblastoma is the most aggressive and lethal primary brain tumor, with a dismal prognosis despite advances in treatment modalities. Bevacizumab, an anti-angiogenic agent, has shown limited efficacy in glioblastoma patients. A study explored miR-21 and miR-10b expression dynamics in response to hypoxia and their potential as biomarkers for bevacizumab response in glioblastoma patients. *In vitro* experiments exposed glioma cells (A172, U87MG, U251) and human umbilical vein endothelial cells to hypoxic conditions, revealing heightened levels of miR-21 and miR-10b. Notably, manipulating miR-10b expression under hypoxic conditions significantly decreased vascular endothelial growth factor alpha levels, suggesting a regulatory role in angiogenesis 38. Additionally, size exclusion chromatography indicated a shift towards miR-21 and miR-10b exosomal packaging during hypoxia. These findings propose that miR-21 and miR-10b could be valuable biomarkers for assessing treatment response and understanding the mechanistic pathways of hypoxia-induced tumor progression in glioblastoma. Incorporating these miRNAs into the diagnostic and treatment planning processes could help personalize glioblastoma management and improve patient outcomes 39.

### Glioma

Gliomas are primary brain tumors that arise from glial cells, with varying degrees of aggressiveness and limited treatment options. In the context of glioma, miR-155 emerges as a key player, influencing cell viability and apoptosis 39. Research has demonstrated that miR-155 targets JARID2, a member of the Jumonji family of proteins involved in epigenetic regulation. Overexpression of miR-155 in glioma cells leads to decreased JARID2 levels, promoting cell proliferation and survival. Interestingly, the study also underscores the therapeutic potential of Valproic acid (VPA) in glioma treatment 40. VPA, a histone deacetylase inhibitor, has been shown to modulate the miR-155/JARID2 axis, inducing apoptosis in glioma cells. These findings offer insights into leveraging miR-155 as a promising therapeutic target in glioma management. Correlation experiments showed a significant correlation between miR-155 concentration and programmed death-ligand 1 (PD-L1) expression levels in high-grade glioma (p-HGG) tumor tissues. However further research is still needed to discover more interesting relationships between immune checkpoints and miRNA molecules in p-HGG 41. We believe that molecular and epigenetic analysis can be the gold standard for pediatric patients seeking effective immunotherapy 40. Developing targeted therapies that inhibit miR-155 or restore JARID2 function could provide novel treatment strategies for this challenging brain tumor 40.

### AML

AML is a heterogeneous hematological malignancy characterized by the uncontrolled proliferation of immature myeloid cells. Investigations into the aberrant expression of miR-155, Kirsten Rat Sarcoma Viral Oncogene Homolog (KRAS), and cAMP Response Element-Binding Protein (CREB) in AML patients have provided valuable insights into the molecular landscape of this blood cancer 42. A study revealed significant upregulation of miR-155 and CREB in AML patients compared to healthy controls. Moreover, a direct correlation was observed between CREB and KRAS expressions, suggesting a potential regulatory relationship. These findings underscore the possible roles of miR-155, KRAS, and CREB in leukemogenesis and highlight their importance as molecular biomarkers in AML. Further research is needed to elucidate the precise mechanisms by which these molecules contribute to AML pathogenesis and to explore their potential as therapeutic targets. Understanding the complex interplay between miRNAs and oncogenic pathways in AML could lead to the development of more targeted and effective treatment strategies 42. The identification of specific targets and pathways regulated by these miRNAs, such as Rho family GTPase 2 (RhoB), PDCD4, FZD5, PI3K/AKT/mTOR, Claudin-1, and PD-L1, provides a foundation for the development of targeted therapies 43. Further elucidation of the intricate molecular mechanisms involving miR-21, miR-34, and miR-155 offers promising avenues for developing targeted therapies and improving patient outcomes. Researchers can identify novel therapeutic strategies that exploit these relationships by understanding the complex networks of miRNA-target interactions and their downstream effects on oncogenic pathways.

## The Complexities of Withdrawn miRNAs in Oncogenesis

**Figure 1:**
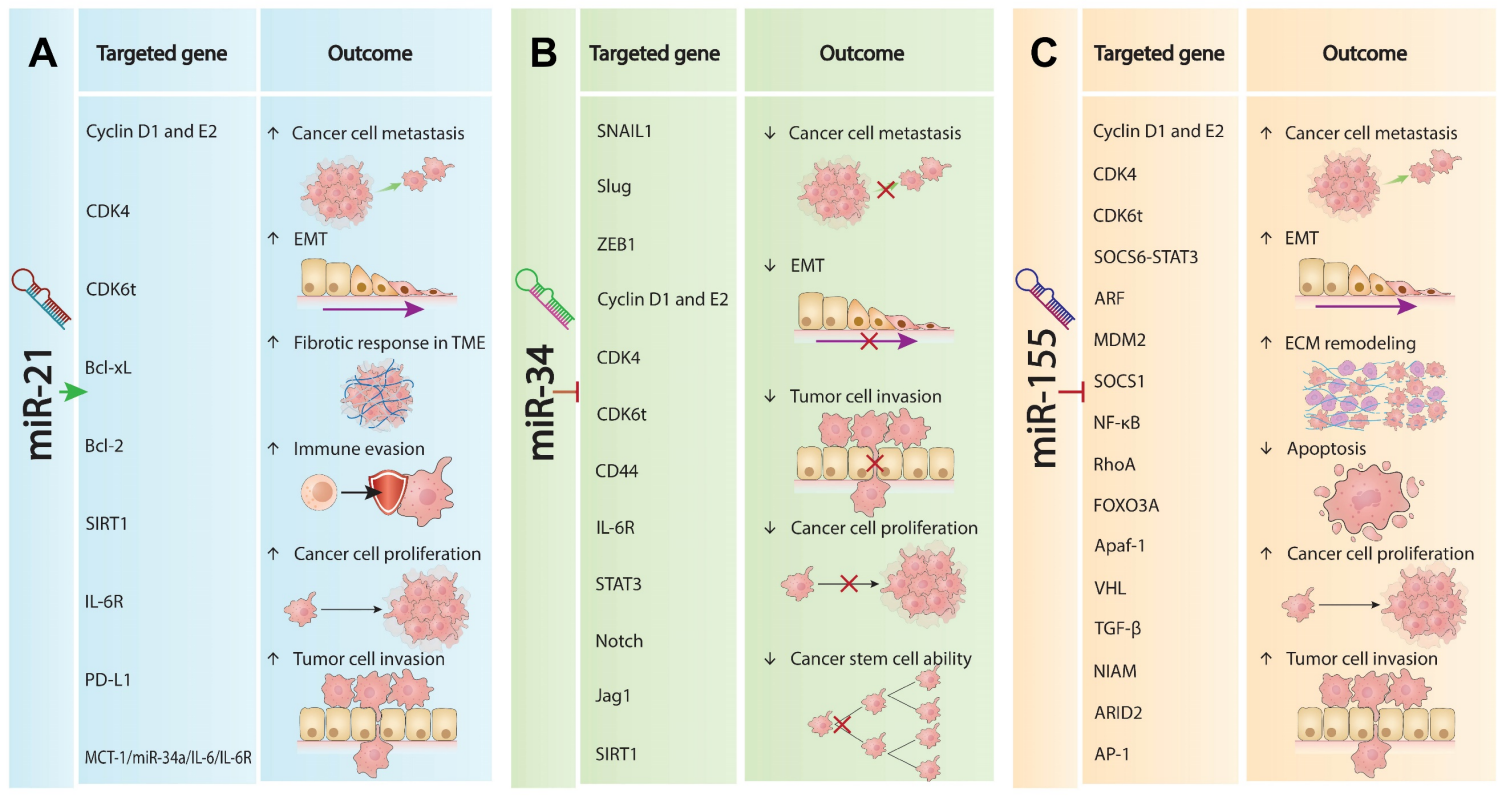
** Roles of withdrawn miRNA in cancer progression. (A).** miR-21 oncogenic mechanisms. miR-21 promotes tumorigenesis by enhancing EMT, increasing metastatic potential and invasiveness. It augments the fibrotic response within the TME, facilitating growth and survival. miR-21 upregulates key oncogenic factors (Cyclin D1, E2, CDK4, CDK6t, Bcl-xL, Bcl-2, SIRT1, IL-6R, PD-L1) that drive proliferation, invasion, metastasis, and immune evasion. Conversely, it downregulates pathways involving MCT-1/miR-34a/IL-6/IL-6R and NRF2, promoting tumor progression. **(B).** miR-34a tumor suppression. miR-34a acts as a tumor suppressor by modulating pathways to inhibit cancer. It upregulates SNAIL1, suppressing EMT and reducing spread and invasion. miR-34a also increases CD44, Cyclin D1, E2, CDK4, IL-6R, and STAT3 levels, decreasing proliferation and growth. Additionally, it downregulates Notch, Jag1, and SIRT1, impairing cancer stem cell self-renewal and suppressing tumor development. **(C).** miR-155 oncogenic properties. miR-155 promotes cancer progression by upregulating Cyclin D1, E2, CDK4, CDK6t, EMT, and extracellular matrix remodeling, enhancing proliferation, dissemination, and invasiveness. It downregulates tumor-suppressive pathways (SOCS6-STAT3, ARF, MDM2, SOCS1, NF-κB, RhoA, FOXO3A, Apaf-1, VHL, TGF-β, NIAM, ARID2, AP-1), leading to increased growth, resistance to tamoxifen, sensitivity to cisplatin, decreased apoptosis, and heightened aggressiveness.

The intricate interplay of distinct roles and molecular impacts of withdrawn miRNAs in oncogenesis sheds light on the complexities underlying cancer progression and the challenges miRNA-based therapies face in clinical settings. Table 1 compares the distinct roles and targeted genes and pathways of all three withdrawn miRNAs in oncogenesis, detailing their impacts on various molecular pathways that underpin cancer progression. Although these miRNAs are crucial in cancer biology and offer promising therapeutic targets in multiple cancers, clinical results for miRNA-based therapies have disappointed 5. miR-21 is a typical oncomiRNA that promotes tumorigenesis by targeting and inhibiting phosphatase and tensin homolog (PTEN), TGF-β, and PDCD4, which in turn activates the PTEN/AKT pathways and EMT 44, 45. This promotes metastasis, invasiveness, and fibrotic responses In the TEM, enhancing tumor growth and survival. Conversely, it downregulates pathways like monocarboxylate transporter 1 (MCT-1)/miR-34a/ interleukin (IL)-6/IL-6 receptor (IL-6R) and nuclear factor erythroid 2-related factor 2 (NRF2), which are crucial for limiting cancer stem cell activity and EMT (Figure 1A). Overall, miR-21 promotes tumor progression by modulating these key pathways and factors 46, 47.

In contrast, miR-34a is a well-established but complex tumor suppressor miRNA that modulates several pathways to inhibit cancer progression 48-51. As shown in Figure 1B, miR-21 upregulates factors such as cluster of differentiation 44 (CD44), Cyclin D1, Cyclin-dependent kinase 4 (CDK4), IL-6R, and signal transducer and activator of transcription 3 (STAT3). The main pathways and genes affected by miR-34a include p53, PI3K/AKT, MAPK, Wnt/β-catenin, MCT-1/miR-34a/IL-6/IL-6R, and reactive oxygen species/nuclear factor erythroid 2-related factor 2 (ROS/KEAP1/NRF2) 49, 52, 53. In many cancers, miR-34a helps maintain cellular balance by regulating pathways that inhibit abnormal cell growth and protect genomic integrity, leading to the suppression of EMT and reduced cancer cell spread and invasion 49. Additionally, miR-34a downregulates Notch, Jagged1 (Jag1), and Sirtuin 1 (SIRT1), impairing the self-renewal capacity of cancer stem cells 15, 16. However, in some cancers, particularly advanced TP53 non-mutant tumors, miR-34a is not consistently downregulated compared to normal tissues, suggesting it may not always fit the typical profile of a tumor suppressor miRNA 49, 54.

miR-155 is a well-documented oncomiRNA in hematology, known for promoting cancer progression by upregulating key factors such as Cyclin D1, Cyclin E2, CDK4, CDK6, SOCS6-STAT3, NF-κB, and TGF-β 34, 55-57. The inhibitory binding of miR-155 collectively enhances tumor cell proliferation, EMT, and extracellular matrix remodeling (Figure 1C) 58, 59. Recent clinical trial analyses in LUAD and non-small cell lung cancer (NSCLC) have shown that downregulation of miR-155 is associated with increased tumor growth, resistance to tamoxifen, sensitivity to cisplatin, decreased apoptosis, and heightened aggressiveness of NSCLC 60.

## Challenges in miRNA withdrawals

### Expression variability

The variability in miRNA expression significantly impacts the effectiveness of miRNA-based therapies. Successful treatments often target miRNAs with consistent expression profiles in specific cancers. For instance, therapies that enhance the expression of tumor-suppressive miRNAs like let-7 have shown promise in cancers where these miRNAs are downregulated 61. Conversely, while miR-21 is typically overexpressed in various cancers, therapies aimed at inhibiting it have faced withdrawal due to adverse effects in patients, underscoring the risks of targeting miRNAs with variable expression 46. In contrast, miR-34, which is often downregulated in many cancers, has demonstrated success in therapies designed to enhance its expression, resulting in more predictable therapeutic outcomes. Thus, understanding and addressing the expression variability of miRNAs is crucial for refining therapeutic strategies and improving patient outcomes.

**Figure 2:**
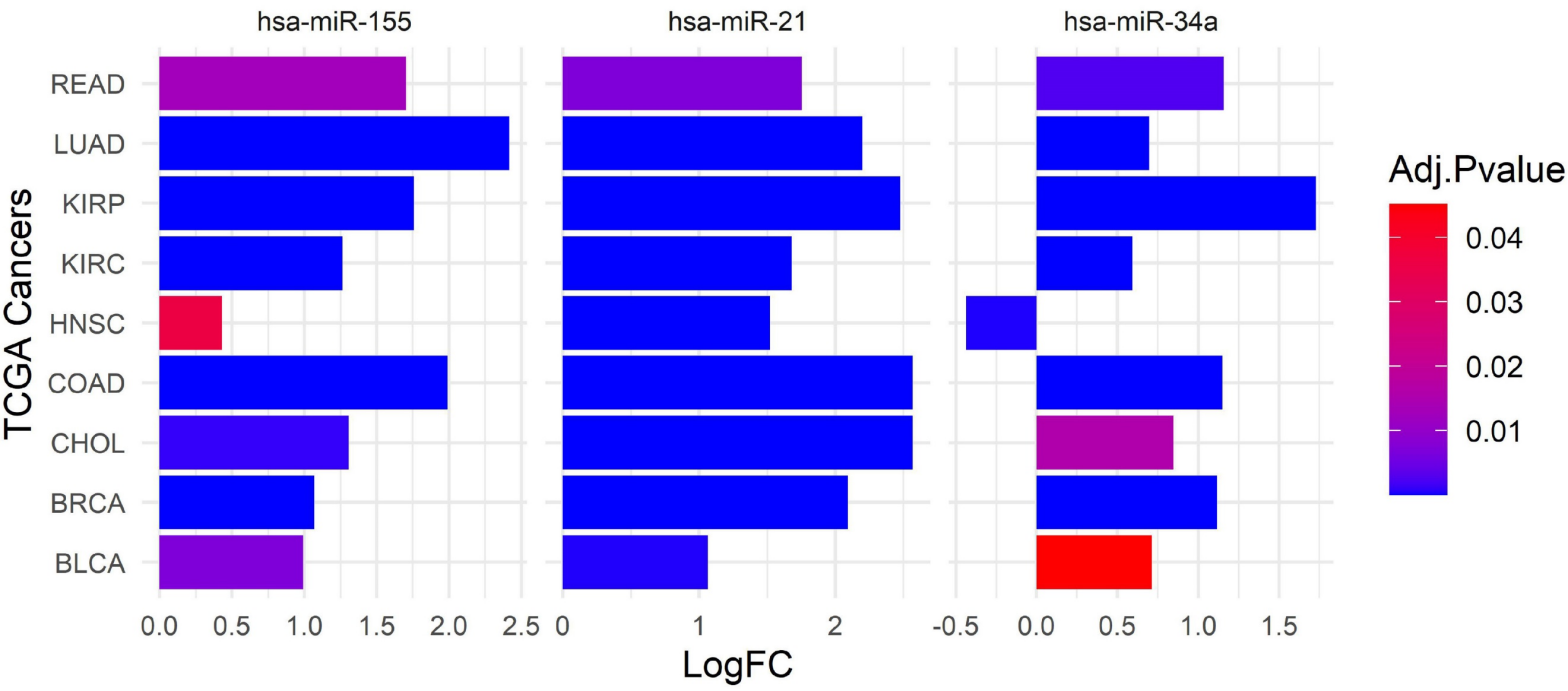
** The expression of withdrawn miRNAs across various TCGA cancers.** This bar plot highlights the differential expression of hsa-miR-155, hsa-miR-21, and hsa-miR-34a across various cancer types, with varying levels of statistical significance. The ubiquitous upregulation of hsa-miR-21 across all studied cancers suggests its broad role in cancer biology, whereas the specific patterns of hsa-miR-155 and hsa-miR-34a provide insights into their unique contributions to certain cancer types. The cancer types evaluated include READ, LUAD, KIRP, KIRC, Head and Neck Squamous Cell Carcinoma (HNSC), COAD, CHOL, BRCA, and BLCA. The expression levels are represented as LogFC on the x-axis, with positive values indicating upregulation and negative values indicating downregulation. We used the dbDEMC 3.0 database (https://www.biosino.org/dbDEMC/index) 62 to extract the LogFC of hsa-miR-21, hsa-miR-34a, and hsa-miR-155, across various cancers.

The differential expression of miRNAs across various cancer types, as evidenced by data from The Cancer Genome Atlas (TCGA), highlights significant variability in miRNA profiles and their implications for therapeutic targeting. Dysregulation of withdrawn miRNAs has been associated with increased tumor aggressiveness, metastasis, and resistance to treatment, highlighting the need for personalized therapeutic strategies and careful patient stratification. To explore this variability, we utilized the dataset from the Database of Differentially Expressed MiRNAs in Human Cancers (dbDEMC) 62, 63, which aggregates high-throughput and low-throughput data to catalog miRNAs exhibiting differential expression in cancers. These miRNAs' Log Fold Change (LogFC) values were extracted and visualized using a bar plot (Figure 2), revealing critical insights into their expression patterns.

Our analysis shows significant expression levels and LogFC values of miR-21 primarily in LUAD, Cholangiocarcinoma (CHOL), Breast Invasive Carcinoma (BRCA), and Bladder Urothelial Carcinoma (BLCA). These findings suggest that miR-21 may contribute to tumor progression and could serve as a potential oncomiR for therapeutic targeting. However, the relatively lower upregulation of miR-21 in Rectum Adenocarcinoma (READ) highlights the complexity of its role in different tumor contexts, potentially reflecting tumor-specific regulatory mechanisms or varying levels of miRNA involvement in tumorigenesis 50, 64, 65.

In contrast, miR-34a demonstrates a complex expression profile. Although it is upregulated compared to miR-34b/c across many human tissues, with peak levels in the brain 66, its expression is notably increased in cancers such as READ, LUAD, CHOL, BRCA, and BLCA. The expression changes are most significant in READ, CHOL, and BLCA 67.

Similarly, miR-155 exhibits variable expression patterns across a range of cancers, including breast, lung, colorectal, pancreatic malignancies, and various hematological cancers 51, 68. Although miR-155 is upregulated in several cancers, such as LUAD, KIRP, KIRC, COAD, CHOL, BRCA, and BLCA, the statistical significance of these changes varies. The variability in miRNA expression presents several challenges for developing effective miRNA-based therapies. It necessitates a comprehensive understanding of miRNA profiles and the factors influencing their expression across different cancer types. Pan-cancer analyses and longitudinal studies are essential to elucidate these miRNAs' overall functions and regulatory networks in cancer. Furthermore, it is crucial to account for the tumor-specific contexts and molecular mechanisms influencing miRNA expression and function.

### Functional Complexity

The functional complexity of miRNAs is another critical factor influencing the success or withdrawal of miRNA-based therapies. Successful interventions often exploit well-characterized miRNAs with defined roles in specific pathways 5, 69. For example, therapies targeting miR-34, known for its role in promoting apoptosis and suppressing oncogenesis, have shown effectiveness in clinical trials. In contrast, therapies that have been withdrawn frequently target miRNAs with broader regulatory roles, leading to unintended consequences. For instance, a therapeutic aimed at inhibiting miR-155, which is involved in both immune regulation and cancer progression, faced challenges due to its complex interplay with multiple biological processes 51.

Off-target effects of miRNAs present significant challenges in evaluating their therapeutic potential. These effects arise when miRNAs interact with unintended target mRNAs, leading to unexpected biological outcomes that can impact the treatment effectiveness and safety. Addressing these issues requires improved targeting strategies and experimental methodologies. A hierarchical clustering heatmap was utilized to analyze the relationships between different isoforms of miR-21, miR-34, and miR-155 and various biological pathways and cancer types. Supplementary File 1 provides detailed interactions between these miRNAs and their target mRNAs, revealing complex regulatory networks.

**Figure 3:**
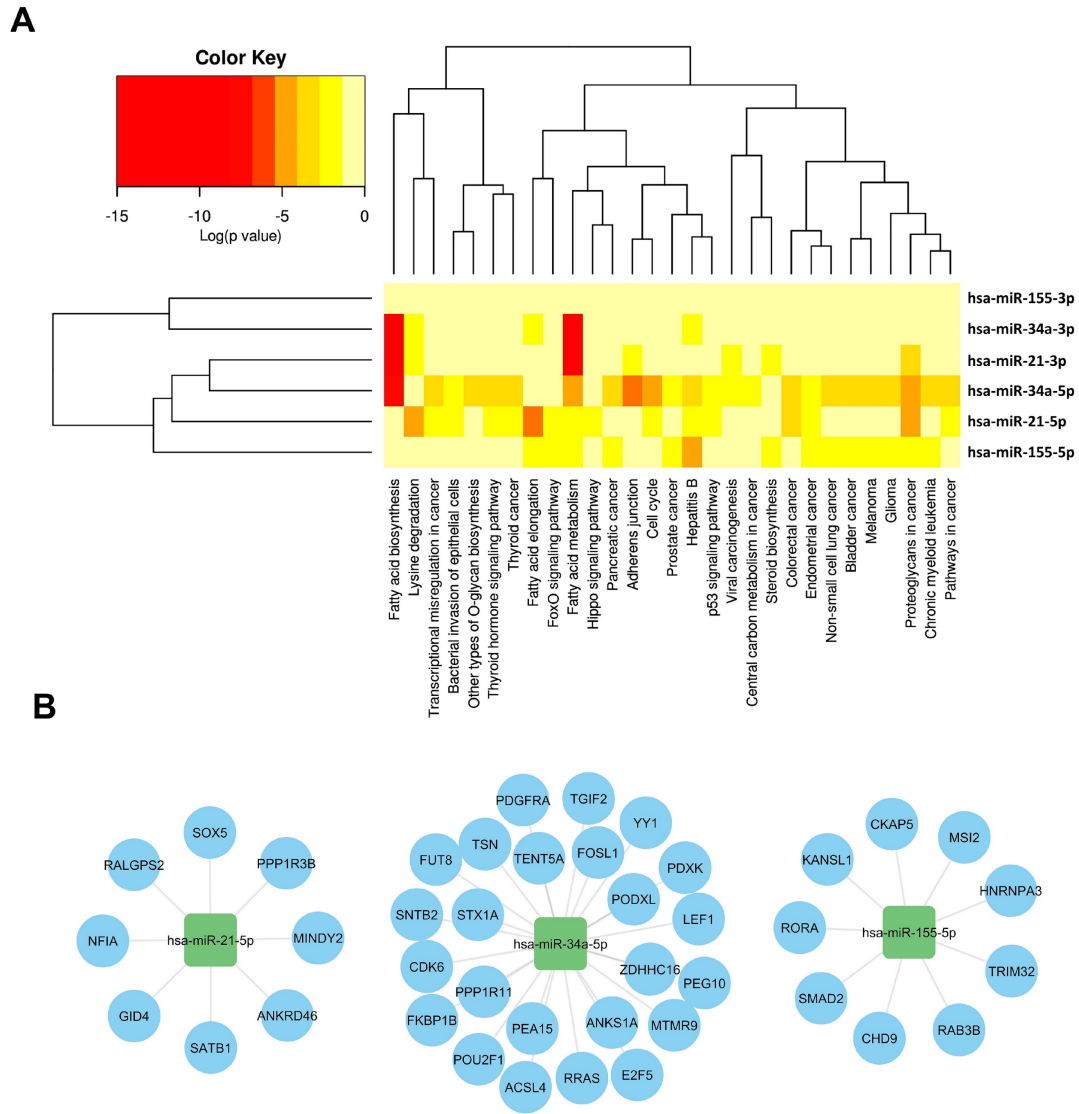
** Regulatory networks and pathway analysis of withdrawn miRNA in cancer.** This figure integrates computational tools to elucidate the regulatory networks and pathway associations involving withdrawn miRNAs, offering a comprehensive view of their impact on cancer biology. **(A).** HCLUST of pathways affected by withdrawn miRNA, using mirPath v.3 (https://dianalab.e-ce.uth.gr/html/mirpathv3/index.php?r=mirpath) 119, reveals clusters influenced by multiple miRNAs, highlighting their overlapping regulatory impact in cancer. Pathways enriched for target genes of withdrawn miRNAs, identified through ToppGene (Supplementary Files 1) 120, provide insights into biological processes and pathways influenced by these miRNAs, emphasizing their significant roles in cancer pathogenesis. **(B).** A bipartite mRNA-miRNA network, derived from miRWalk 3.0 121, illustrates the complex regulatory relationships between withdrawn miRNAs (squares) and their specific target genes (circles). This network structure underscores the multifaceted regulatory roles of miRNAs in cancer biology, highlighting their intricate interactions and regulatory significance.

As illustrated in Figure 3, As illustrated in Figure 3, miR-21, miR-34, and miR-155 each exhibit unique functional complexities. MiR-34a is involved in regulating cell cycle progression, apoptosis, and metastasis (Figure 3A). It targets regulators such as Cyclins D1 and E2, CDK4, and CDK6, inducing cell cycle arrest at the G1 phase and promoting apoptosis by modulating anti-apoptotic and pro-apoptotic proteins 74-80. MiR-34a also suppresses angiogenesis by targeting VEGF and its receptors and downregulates SIRT1, enhancing p53 activation to promote cellular senescence and growth arrest 81-86. Furthermore, miR-34a downregulates Notch, Jag1, and SIRT1, impairing cancer stem cell self-renewal and reducing cancer cell invasiveness 70-75.

MiR-21, shown in Figure 3B, targets crucial cell cycle and apoptosis regulators such as Cyclin D1, Cyclin E2, CDK4, CDK6, Bcl-xL, and Bcl-2 76-78. It influences cell proliferation, survival, and resistance to apoptosis, notably affecting the PI3K/AKT pathway by downregulating PTEN, which leads to increased AKT activity and enhanced cell growth and survival 79,65, 66. MiR-21 also impacts the RAS/MAPK pathway and induces EMT through modulation of TGF-β and PI3K/AKT pathways 80, 81.

MiR-155's diverse roles in immune modulation 82, inflammation 83, and oncogenesis 84 further complicate its therapeutic application (Figure 3C). It affects immune responses within the TME by influencing the balance between pro-inflammatory and anti-inflammatory signals and regulates various immune system components including T cells, macrophages, and dendritic cells 85, 86. Its involvement in these diverse processes underscores the complexity of targeting miR-155 and highlights the need for careful consideration of tumor-specific contexts in therapeutic development.

The multifaceted roles and off-target effects of miR-21, miR-34, and miR-155, as summarized in Figure 3, emphasize the need for precise targeting strategies and thorough evaluation. These insights are critical for refining miRNA-based therapies and improving their clinical outcomes, guiding the development of more effective and safer treatment approaches. The complexities encountered with these miRNAs underscore the importance of targeted strategies and comprehensive evaluation in ensuring therapeutic efficacy and safety.

### Clinical Implications

The implications of miRNA therapy withdrawals extend beyond individual patients, influencing broader clinical practices and research trajectories. Successful therapies contribute to our understanding of miRNA mechanisms and help establish evidence-based guidelines for future treatments 87. For instance, the successful clinical application of miRNA mimics has paved the way for developing similar strategies in other contexts. Conversely, the withdrawal of certain miRNA therapies highlights critical lessons regarding safety and efficacy assessment 88. The case of a withdrawn miRNA inhibitor, which demonstrated promise in preclinical studies but failed to translate in clinical trials due to safety concerns, serves as a cautionary tale 5. This comparative analysis of successful and withdrawn therapies not only identifies the factors that underpin the efficacy and safety of miRNA-based interventions but also emphasizes the need for robust preclinical evaluations and adaptive trial designs 89. By learning from these experiences, researchers can enhance the development of safer, more effective miRNA therapeutics.

**Figure 4:**
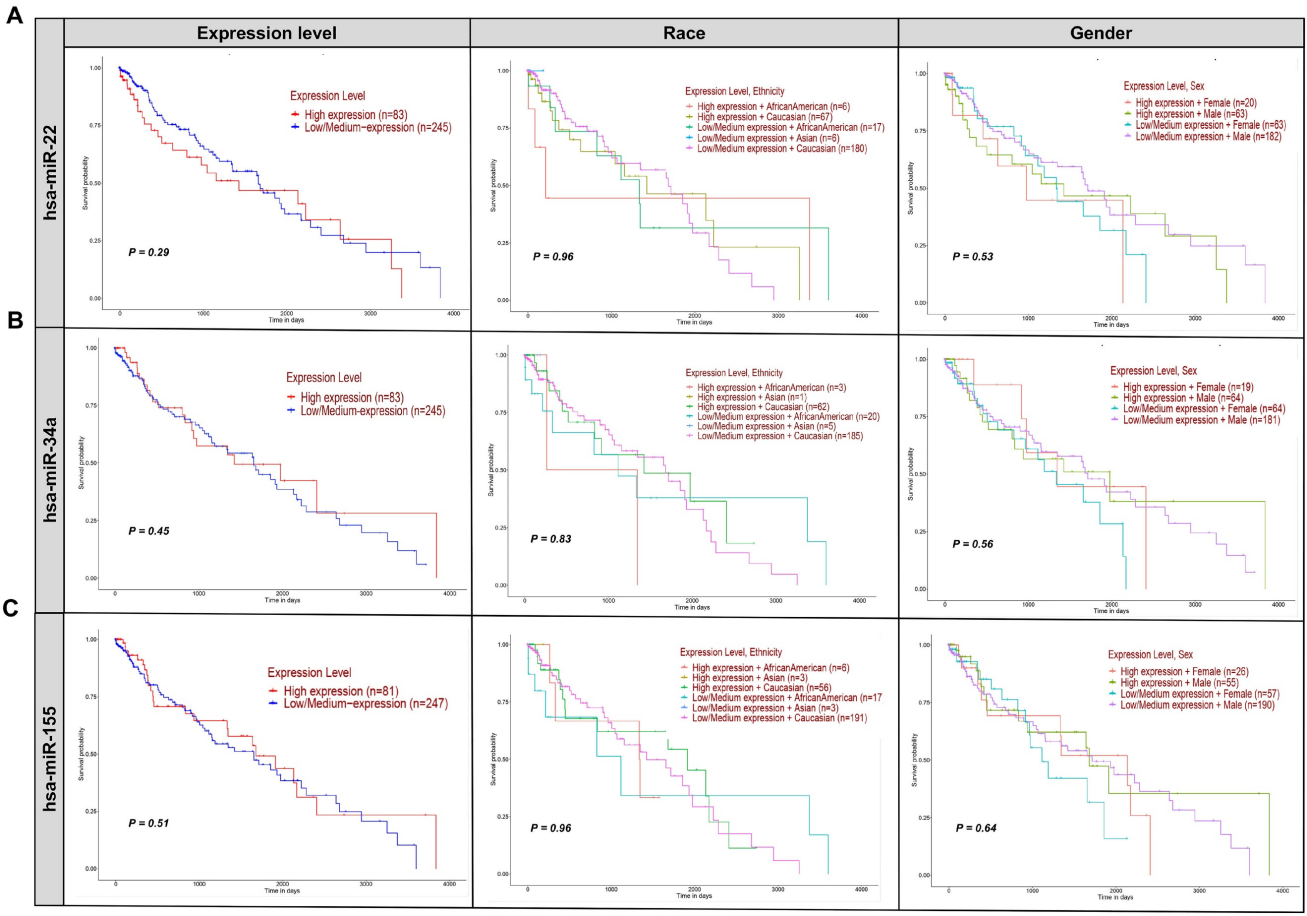
** Effect of withdrawn miRNA expression on LUSC patient survival.** This figure presents Kaplan-Meier survival plots analyzing the impact of hsa-miR-22 **(A)**, hsa-miR-34a **(B)**, and hsa-miR-155 **(C)** expression levels on the survival of patients with LUSC, based on gender, race, and expression levels. The lack of significant differences (p-values > 0.05) across all comparisons suggests that these miRNAs do not play a determinative role in altering patient survival outcomes in this context. The expression levels of hsa-miR-22, hsa-miR-34a, and hsa-miR-155, whether considered alone or in combination with race or sex, do not significantly impact the survival of LUSC patients. Each row of plots corresponds to one miRNA, and each column represents different stratifications: expression level only, expression level and race, and expression level and sex. All patient survival information for the withdrawn miRNA was analyzed using OMICS data from the TCGA dataset for all three miRNAs via the UALCAN (https://ualcan.path.uab.edu/index.html) platform 90.

Kaplan-Meier survival plots were employed to assess the impact of miR-22, hsa-miR-34a, and hsa-miR-155 expression levels on the survival of patients with LUSC, a cancer type characterized by high expression of these miRNAs. Supplementary File 2 presents the enrichment analysis of diseases linked to the withdrawn miRNAs extracted from ToppGene. This file highlights how these miRNAs are associated with a variety of cancers and other diseases (Figure 4) 90.

Figure 4A shows Kaplan-Meier survival plots for miR-22, indicating no significant difference in survival between high-expression (n=49) and low-expression (n=245) groups (p=0.29). Analyses considering expression levels along with race and sex also showed no significant interactions (p=0.98 and p=0.53, respectively). These results suggest that hsa-miR-22 expression levels do not significantly affect the survival of lung squamous cell carcinoma (LUSC) patients. Regarding hsa-miR-21, while its levels are associated with cancer progression, its therapeutic potential remains unclear. Translating this biomarker potential into effective treatments requires further investigation into its molecular mechanisms and clinical implications 91, 92. A Phase III trial (NCT06015815||https://www.clinicaltrials.gov/search?cond=NCT06015815) was requested to explore miR-21's impact on NSCLC. The trial, which included 21 participants over 10 months, aimed to assess various aspects of miR-21, such as performance status, patient symptoms, and miRNA levels, particularly about acute side effects of chemoradiotherapy 93, 94. Despite its promise, the trial results did not meet anticipated endpoints, highlighting the challenges in realizing miR-21's therapeutic potential. The study underscores the complexity of miR-21 in cancer biology and the hurdles in translating its promise into clinical practice 95.

MRX34, the first miRNA-based drug, initially generated significant enthusiasm for its potential in cancer therapy (Table 2). Clinical trials, such as NCT01829971, involving 155 participants across various cancers, including liver cancer and solid tumors, examined miR-34a's therapeutic role (NCT01829971||https://clinicaltrials.gov/study/NCT01829971) 54, 91, 92. Despite some adverse immune reactions, MRX34 showed promise, particularly in LUSC, where it inhibited tumor growth and induced regression 96. The trial also explored the co-delivery of miR-34a and let-7b using NOV340, demonstrating efficacy in non-small cell lung cancer (NSCLC) models resistant to conventional treatments 91. However, challenges such as miRNA degradation, immune reactions, and nanoparticle toxicity necessitated a cautious approach, leading to MRX34's withdrawal in the clinical trial NCT02862145 due to immune-related adverse events (NCT02862145||https://clinicaltrials.gov/study/NCT02862145?cond=NCT02862145&rank=1) 84. Despite these setbacks, ongoing and planned trials, like NCT01057199, continue to explore miR-34a's potential, focusing on AML through mutation analysis and gene expression profiling (NCT01057199||https://www.clinicaltrials.gov/study/NCT01057199?cond=NCT01057199&rank=1) 66. Survival analysis of miR-34a in LUSC showed no significant difference in survival between high (n=40) and low (n=246) expression groups (p=0.45) (Figure 4B). Stratified analyses by race and sex also revealed no significant interactions (p=0.83 and p=0.56, respectively), suggesting that miR-34a may not be a reliable clinical marker for predicting survival in LUSC patients. Immune-related adverse events were a major concern during the MRX34 trial, with symptoms such as fever and fatigue highlighting the need for rigorous safety assessments in clinical trials 97. Preclinical studies demonstrated miR-34a's potential in inhibiting tumor growth, but translating these results into effective therapies proved challenging due to issues with miRNA degradation, immune reactions, and delivery system toxicity 98. The complexity of miR-34a's molecular interactions and its variable expression across different cancers further complicates its therapeutic application 99. Variability in survival rates and regulatory mechanisms across cancer types underscores the need for a nuanced approach to developing miR-34a-based treatments (see Figure 2 100).

The miR-155-5p, a key member of the miR-155 family, has been extensively studied for its potential to improve cancer outcomes in clinical settings (Figure 4C) 91. However, its withdrawal from clinical trials underscores the difficulties in translating preclinical findings into effective therapies. Trials such as NCT03591367 (NCT03591367||https://clinicaltrials.gov/study/NCT03591367?cond=NCT03591367%20&rank=1) and NCT06015815 (NCT06015815||https://clinicaltrials.gov/study/NCT06015815?cond=NCT06015815%20&rank=1) were designed to explore miR-155's impact on BC and NSCLC, respectively 101. The NCT03591367 trial, focusing on breast cancer, involved 115 participants and assessed miR-155's effects on sensitivity, specificity, and TERT expression over 9 months. It also examined secondary endpoints such as quality of life and vitamin D levels. Meanwhile, the NCT06015815 trial, a phase III study in NSCLC, included 21 participants and evaluated miR-155's role in ECOG performance status and the acute side effects of chemoradiotherapy over 10 months (NCT06015815|| https://clinicaltrials.gov/study/NCT06015815?cond=NCT06015815%20&rank=1). Despite the comprehensive design of these trials, miR-155's clinical application faced significant challenges, leading to its withdrawal. Analysis in Figure 4C reveals that miR-155 expression levels did not significantly impact survival rates in LUSC patients, regardless of race or gender. This lack of significant findings highlights the complexities involved in translating miR-155's potential into clinical benefits and underscores the need for further research clinically 91. Preclinical studies demonstrate miR-155's anti-tumor potential, but translating these findings into effective clinical therapies presents significant hurdles. Optimizing delivery systems to enhance miR-155 stability, tissue specificity, and efficacy is crucial. Although nanoparticle-based delivery systems have shown promise in preclinical models, issues such as systemic toxicity and immunogenicity must be resolved before advancing to clinical applications 93, 94.

## Prospects

**Figure 5:**
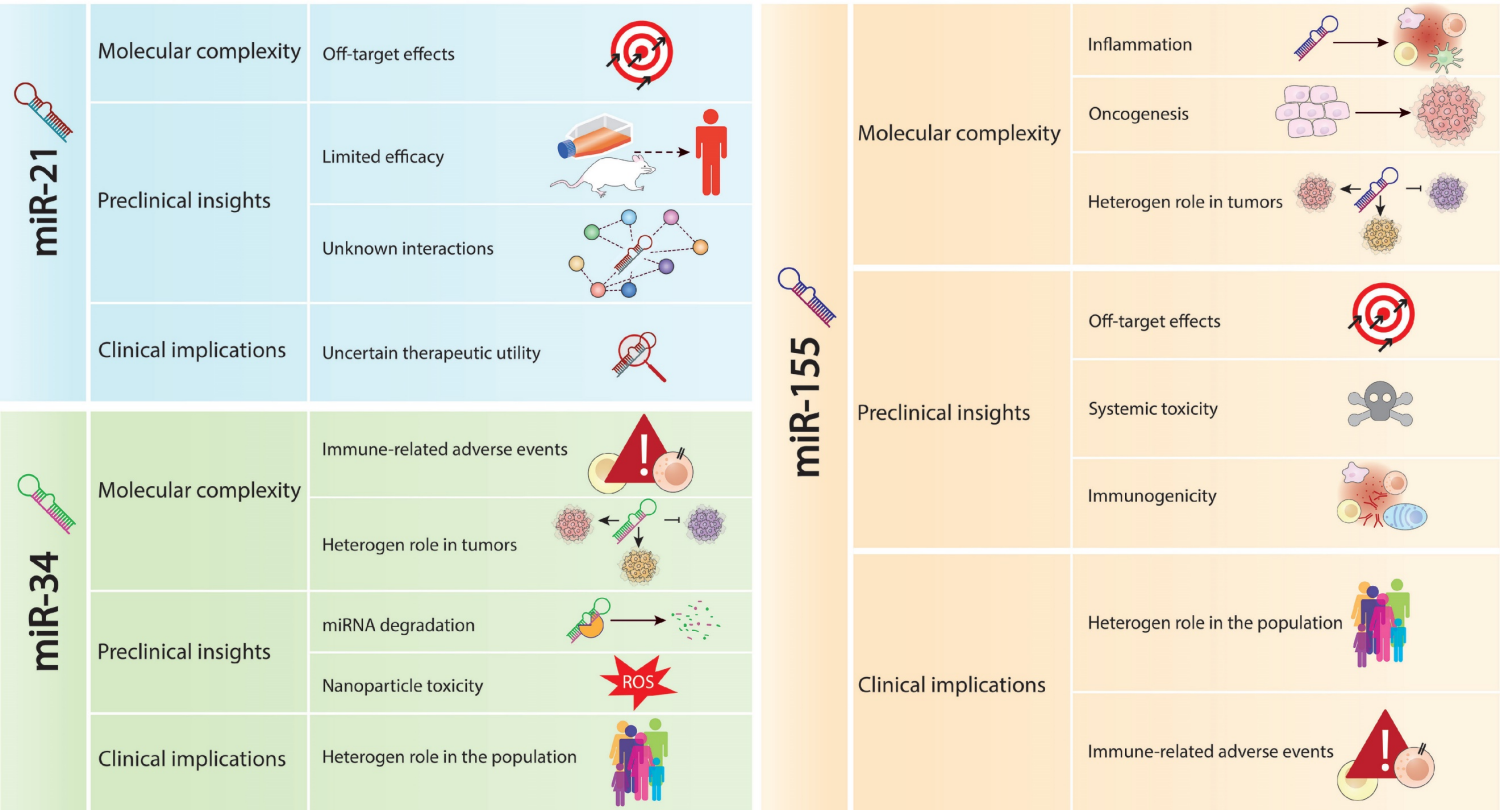
** Challenges associated with the withdrawal of miRNAs from clinical trials.** This figure outlines the main challenges leading to the withdrawal of miRNAs, including miR-21, miR-34a, and miR-155, from clinical trials. The challenges include off-target effects, limited efficacy in preclinical models, inadequate understanding of regulatory networks, and the focus on biomarker potential rather than therapeutic utility for miR-21. For miR-34a, challenges involve immune-related adverse events, preclinical translation hurdles, molecular complexity, and survival rates. miR-155's challenges encompass molecular complexity, clinical implications, and difficulties in translating promising preclinical data into clinical practice. This comprehensive analysis underscores the multifaceted challenges encountered in miRNA-based therapies, impacting their clinical translation and therapeutic efficacy.

Figure 5 highlights key advancements, including improvements in nanoparticle-based delivery systems, personalized medicine approaches guided by biomarkers, and the integration of miRNA therapies with existing and new treatments. Innovations in these areas, along with new research into therapeutic targets, are set to shape the future of miRNA strategies in cancer therapy. These developments aim to enhance treatment outcomes by making therapies more precise and effective and reducing resistance. The following details outline these promising directions for miRNA therapy in cancer treatment.

### Innovative Delivery Systems

New delivery systems are crucial for advancing miRNA strategies in cancer therapy by improving stability, circulation time, and tumor-targeting capabilities, thereby optimizing drug delivery while reducing off-target effects 102, 103. Following the success of COVID-19 treatments, nanoparticle-based platforms, including lipid nanoparticles and polymeric micelles, are being refined to enhance their specificity, efficacy, and safety 104. Emerging delivery vehicles, such as exosomes, extracellular vesicles, and cell-derived nanoparticles, offer advantages like natural biocompatibility and efficient cargo delivery. Additionally, bioengineered viral vectors, such as adeno associated viruses and lentiviral vectors, provide opportunities for high transduction efficiencies and sustained gene expression in target tissues, paving the way for improved therapeutic outcomes 105, 106.

### Combination Therapies

Documentarily, combining miRNA therapy with chemotherapy, radiotherapy, and emerging treatments offers a multifaceted approach to enhance therapeutic efficacy, especially in chemotherapy-resistant tumors 107, 108. Synergistic effects have been observed when miRNA therapies are combined with chemotherapy, radiotherapy, or targeted therapies, potentially boosting anti-tumor responses while reducing adverse effects. Integrating miRNA-based immunotherapies, such as immune checkpoint inhibitors or CAR T-cell therapy, holds promise for leveraging the immune system's potential to achieve double responses 109, 110. Additionally, exploring synthetic lethal interactions between miRNA withdrawals and oncogenic pathways supports the development of precision combination therapies tailored to the specific molecular characteristics of tumors, optimizing treatment outcomes and extending patient survival 111.

### Personalized Approaches

Although miRNA therapy functions at the post-transcriptional level within complex networks involving mRNAs, transcription factors, lncRNAs, circRNAs, and other non-coding RNAs, personalized approaches can transform its application. These strategies aim to tailor therapies to individual patients' unique molecular profiles, leveraging the intricate interactions between miRNA, mRNA, and lncRNAs 100. Personalized miRNA therapies are grounded in biomarker-guided patient stratification. By utilizing genomic profiling, transcriptomic analysis, and specific miRNA expression signatures, clinicians can precisely identify patient subgroups most likely to benefit from targeted therapies 88. This approach ensures that treatments are tailored to the genetic and molecular characteristics of each patient's cancer, potentially increasing efficacy and minimizing adverse effects. Liquid biopsy techniques, including circulating miRNA profiling and extracellular vesicle analysis, offer non-invasive methods for real-time monitoring of treatment responses and disease progression 88. These techniques facilitate timely adjustments to treatment regimens based on the current patient status, enabling a dynamic and responsive approach to cancer care. Furthermore, incorporating patient-derived organoid models and xenografts into preclinical studies enhances the predictive testing of therapeutic efficacy. These models allow for the evaluation of treatment responses in a setting that closely mimics the patient's tumor biology, aiding in the identification of optimal treatment combinations and improving overall treatment outcomes 88, 112. Using the basket trials, which focus on molecular alterations rather than tumor histology, enables the evaluation of miRNA therapies across different cancer types and helps identify patient subgroups likely to benefit from these treatments 113. Standardizing these methods across trials is essential for translating findings from preclinical to clinical settings, ultimately unlocking the potential of miRNA-based interventions for personalized and effective cancer treatment 111.

### Advancements in Prognostic and Therapeutic Targets

Identifying and validating new prognostic and therapeutic biomarkers for miRNA-based therapies is crucial for expanding treatment options and improving outcomes. High-throughput screening methods, including network analysis, pathway mapping, CRISPR/Cas9-based functional genomics, and small molecule libraries, enable the systematic exploration of miRNA-target interactions, identifying potential drug targets 110, 114. Additionally, computational modeling and artificial intelligence algorithms offer predictive insights into miRNA-regulated networks, guiding the development of targeted therapies and new biomarkers 88. Advances in biomarker development and validation are essential for optimizing patient selection, monitoring treatment responses, and guiding therapeutic decisions in miRNA-based therapies. Integrating multi-omics data-encompassing genomics, transcriptomics, proteomics, and metabolomics provides a comprehensive tumor profile and helps identify predictive biomarker signatures 115.

Machine learning algorithms and bioinformatics tools facilitate the integration of diverse datasets and the development of robust predictive models for patient stratification 116, 117. These technologies accelerate cancer drug development, including miRNA-based therapies, by streamlining the testing, validation, and synthesis processes. Advances in computational technology and multi-omics data have led to innovative bioinformatics, pharmacoinformatics, and chemoinformatics tools, which significantly enhance the efficiency of miRNA-based therapy development. Longitudinal monitoring of dynamic biomarkers, such as circulating miRNA levels, tumor-derived exosomes, and radiomic features, offers insights into treatment response and disease progression, providing valuable information for optimizing treatment and patient management 116, 117. Establishing standardized protocols for biomarker validation and clinical implementation will accelerate the translation of these discoveries into clinical practice, improving patient outcomes in miRNA-based therapies 118.

## Conclusion

This review underscores the importance of miR-21, miR-34, and miR-155 in cancer pathogenesis and highlights their potential as biomarkers and therapeutic targets. The ongoing efforts to unravel the multifaceted roles of miRNAs in cancer biology hold great promise for advancing personalized medicine and improving patient care. Further research into the mechanistic underpinnings of these miRNAs and their interactions with other molecular players will pave the way for developing novel, targeted therapies that can revolutionize cancer treatment and improve patient outcomes across a wide range of malignancies. Furthermore, this review addresses the reasons behind the withdrawal of miRNA-based therapies for solid cancers. We explore key issues such as expression variability, off-target effects, limitations in drug delivery, and functional complexity. By highlighting these factors, we aim to guide future research and underscore the potential of miRNA therapies in targeting cancer pathways and enhancing anti-tumor responses. With ongoing advancements in delivery methods, combination therapies, and personalized treatment approaches, miRNA therapies hold promise for improving patient outcomes and advancing precision cancer therapy.

## Supplementary Material

Supplementary file 1.

Supplementary file 2.

## Figures and Tables

**Table 1:** Functional targets and pathways of withdrawn miRNAs in cancer biology.

miRNA	Cancer	Expression	Target genes	Mechanism of action	Pathways	References
miR-34a	GC	UP	Cyclin D1, Cyclin E2, CDK4, CDK6	Promotes inhibition of cancer-inhibiting processes, inhibiting proliferation, invasion, and metastasis	p53, PI3K/AKT, MAPK, Wnt/β-catenin	55
	BC	DOWN	Bcl-xL, Bcl-2, SIRT1, IL-6R	Enhances cancer cell invasion and metastasis.	p53, Wnt/β-catenin, MCT-1/miR-34a/IL-6/IL-6R	49, 52
	TNBC	UP	Stat3, IL-6R	Inhibits cancer stem cells and EMT progression.	MCT-1/miR-34a/IL-6/IL-6R	52
	CRC	UP	IL-6R, NRF2	Promotes tumor cell invasion and metastasis.	MCT-1/miR-34a/IL-6/IL-6R, ROS/KEAP1/NRF2	53
miR-21	NSCLC	UP	PDCD4, PTEN, RECK	Inhibiting its expression can induce tumor cell apoptosis.	PTEN, TGF-β, PDCD4	26
	CRC	UP	PTEN, PDCD4, RECK, STAT3	Enhances tumor aggressiveness, invasion, and metastasis; Increases sensitivity to treatments; Alters cell cycle and drug response.	TGF-β	50, 65
	GC	UP	PTEN	Enhances gastric cancer cell differentiation, angiogenesis, and metastasis	PTEN	79
	PCa	UP	PDCD4, PTEN	Inhibits pro-apoptotic signals.	PTEN/AKT	44
miR-155	BC	UP	SOCS6, STAT3, ARF, MDM2, SOCS1, NF-κB, RhoA, FOXO3A	Promotes tumor growth, mediates tamoxifen resistance, alters therapy sensitivity, influences invasiveness, and modulates energy metabolism.	SOCS6-STAT3, JAK-STAT, EMT, PI3K-AKT	34, 56, 57
	LC	UP	SOCS6, STAT3, Apaf-1, VHL, TGF-β, RhoA	Reduces cisplatin sensitivity, enhances tamoxifen resistance, and increases invasiveness.	SOCS6/STAT3, Apaf-1, TGF-β, RhoA	33, 34
	BCL	UP	ARID2, AP-1, NF-κB	Promotes tumor growth.	NIAM	34
	HCC	UP	Cyclin D1, Cyclin E2, CDK4, CDK6	Promotes inhibition of cancer-inhibiting processes, inhibiting proliferation, invasion, and metastasis	SOCS1/STAT3/NF-κB	55

Abbreviation list: AP-1, Activator Protein 1; Apaf-1, Apoptotic Protease Activating Factor 1; ARF, Alternate Reading Frame; ARID2, AT-Rich Interaction Domain 2; BC, Breast Cancer; Bcl-xL, B-cell lymphoma-extra large; cMYC, MYC Proto-Oncogene, BHLH Transcription Factor; CRC, Colorectal Cancer; DOWN, Downregulated; EMT, Epithelial-Mesenchymal Transition; FOXO3A, Forkhead Box O3; GC, Gastric Cancer; HCC, Hepatocellular Carcinoma; IL-6, Interleukin-6; IL-6R, Interleukin-6 Receptor; IL-6R, Interleukin-6 Receptor; KEAP1, Kelch-like ECH-Associated Protein 1; MAPK, Mitogen-Activated Protein Kinase; MCT-1, Monocarboxylate Transporter 1; MDM2, Mouse Double Minute 2 Homolog; NF-κB, Nuclear Factor Kappa B; NRF2, Nuclear Factor Erythroid 2-Related Factor 2; NSCLC, Non-Small Cell Lung Cancer; PCa, Prostate Cancer; PDCD4, Programmed Cell Death 4; PDK, Pyruvate Dehydrogenase Kinase; PI3K/AKT, Phosphoinositide 3-Kinase/Protein Kinase B; PIK3R1, Phosphoinositide-3-Kinase Regulatory Subunit 1; PTEN, Phosphatase and Tensin Homolog; RECK, Reversion-Inducing Cysteine-Rich Protein with Kazal Motifs; RhoA, Ras Homolog Family Member A; ROS, Reactive Oxygen Species; SIRT1, Sirtuin 1; SOCS6, Suppressor of Cytokine Signaling 6; STAT3, Signal Transducer and Activator of Transcription 3; TGF-β, Transforming Growth Factor Beta; TNBC, Triple-Negative Breast Cancer; UP, Upregulated; VHL, Von Hippel-Lindau Tumor Suppressor; Wnt/β-catenin, Wingless/Integrated.

**Table 2:** Overview of withdrawn clinical trials on miRNA therapy in cancer.

miRNA	NCT No.	Cancer type	TNM	Sample size (F/M)	Eligibility	Country	Other details	Clinical trial references
miR-21	NCT06015815	NSCLC	III	21	-Age: 18-85 yrs -Gender:All	TR	- Duration: 10 mos. - Primary Endpoint: ECOG, PS, miRNA levels with acute side effects of chemoradiotherapy - Secondary Endpoints: CTCAE, - Measurement Method: RT-qPCR	https://www.clinicaltrials.gov/search?cond=NCT06015815
miR-34a	NCT01829971	LC	IV	50 (20/30)	- Age: 40-75 yrs - Gender: Male (60%), Female (40%)	CN	- Duration: 49 mos. - Primary Endpoint: MTD, RPh2D - Secondary Endpoints: Peak blood concentration, AUC - Measurement Method: Intravenous administration of MRX34.	https://clinicaltrials.gov/study/NCT01829971 https://www.clinicaltrials.gov/search?cond=NCT06015815
	NCT01057199	AML	NA	6	- Ages Eligible for Study: Child, Adult, Older Adult Gender: All	USA	- Duration: 6 mos. - Primary Endpoint: C/EBPα, E2F3 - Secondary Endpoints: NA -Measurement Method: Mutation analysis, gene expression analysis, RNA analysis.	
miR-155	NCT03591367	BC	NA	115	- Age: 18 yrs and older - Gender: All	EG	- Duration: 9 mos. - Primary Endpoint: Sensitivity, specificity, positive and negative predictive values, TERT - Secondary Endpoints: QOL, Vitamin D - Measurement Method: RT-qPCR	https://clinicaltrials.gov/study/NCT03591367?cond=NCT03591367%20&rank=1
	NCT06015815	NSCLC	III	21	-Age: 18-85 yrs -Gender:All	TR	- Duration: 10 mos. - Primary Endpoint: ECOG, PS, miRNA levels with acute side effects of chemoradiotherapy - Secondary Endpoints: CTCAE, NA - Measurement Method: RT-qPCR	https://www.clinicaltrials.gov/search?cond=NCT06015815
miR-16	NCT02369198	MPM, NSCLC	Ⅰ	27	- Age: 18-95 yrs - Gender: All	AU	- Duration: 28 months - Primary Endpoint: MTD, PS, dose-limiting toxicities - Secondary Endpoints: QOL, ECOG, PS. - Measurement Method: Planned dose levels.	https://clinicaltrials.gov/study/NCT02369198?cond=NCT02369198%20&rank=1

Abbreviation list: AML, Acute Myeloid Leukemia; AU, Australia; AUC, Area Under the Curve; BC, Breast Cancer; CTCAE, Common Terminology Criteria for Adverse Events; ECOG, Eastern Cooperative Oncology Group; EG, Egypt; F/M, Female/Male; IV, Stage IV; LC, Lung Cancer; MPM, Malignant Pleural Mesothelioma; MTD, Maximum Tolerated Dose; NSCLC, Non-Small Cell Lung Cancer; PS, Performance Status; QOL, Quality of Life; RPh2D, Recommended Phase 2 Dose; RT-qPCR, Reverse Transcription Quantitative Polymerase Chain Reaction; TNM, Tumor-Node-Metastasis.

## References

[1] Chivukula RR, Mendell JT (2008). Circular reasoning: microRNAs and cell-cycle control. Trends Biochem Sci.

[2] Mens MMJ, Ghanbari M (2018). Cell Cycle Regulation of Stem Cells by MicroRNAs. Stem Cell Rev Rep.

[3] Zhang S, Cheng Z, Wang Y, Han T (2021). The Risks of miRNA Therapeutics: In a Drug Target Perspective. Drug Des Devel Ther.

[4] Bader AG (2012). miR-34 - a microRNA replacement therapy is headed to the clinic. Front Genet.

[5] Seyhan AA (2024). Trials and Tribulations of MicroRNA Therapeutics. Int J Mol Sci.

[6] Le DH, Verbeke L, Son LH, Chu DT, Pham VH (2017). Random walks on mutual microRNA-target gene interaction network improve the prediction of disease-associated microRNAs. BMC Bioinformatics.

[7] Arslan Bozdag L, Acik L, Ersoy HE, Bayir O, Korkmaz MH, Mollaoglu N (2024). PDCD4 and MIR-21 are promising biomarkers in the follow-up of OED in liquid biopsies. Oral Dis.

[8] He J, Yu K, Liang G, Shen W, Tian H (2024). circ_0000592 facilitates the progression of esophageal squamous cell carcinoma via miR-155-5p/FZD5 axis. J Biochem Mol Toxicol.

[9] Tomasetti M, Monaco F, Rubini C, Rossato M, De Quattro C, Beltrami C (2024). AGO2-RIP-Seq reveals miR-34/miR-449 cluster targetome in sinonasal cancers. PLoS One.

[10] Charbit H, Lavon I (2024). Investigating Expression Dynamics of miR-21 and miR-10b in Glioblastoma Cells *In Vitro*: Insights into Responses to Hypoxia and Secretion Mechanisms. Int J Mol Sci.

[11] Moreira D, Alexandre D, Miranda A, Lourenco P, Baptista PV, Tomaz C (2024). Detecting mir-155-3p through a Molecular Beacon Bead-Based Assay. Molecules.

[12] Alexandre D, Fernandes AR, Baptista PV, Cruz C (2024). Evaluation of miR-155 silencing using a molecular beacon in human lung adenocarcinoma cell line. Talanta.

[13] Hong Y, Li Z, Su Y, Pu H, Zhang X (2024). The ceRNA Mechanism of lncRNA MEG3/miR-21-5p/SPRY2 in Cell Proliferation and Apoptosis in Bladder Cancer. Crit Rev Eukaryot Gene Expr.

[14] Qiao L, Dong C, Jia W, Sun G (2024). RAB5A in triple-negative breast cancer: a critical role in macrophage reshaping in an exosomal miR-21-dependent manner. Endocr Relat Cancer.

[15] Luo A, Liu H, Huang C, Wei S (2024). Exosome-transmitted circular RNA circ-LMO7 facilitates the progression of osteosarcoma by regulating miR-21-5p/ARHGAP24 axis. Cancer Biol Ther.

[16] Kim WR, Park EG, Lee DH, Lee YJ, Bae WH, Kim HS (2023). The Tumorigenic Role of Circular RNA-MicroRNA Axis in Cancer. Int J Mol Sci.

[17] Yin X, Lin H, Lin L, Miao L, He J, Zhuo Z (2022). LncRNAs and CircRNAs in cancer. MedComm (2020).

[18] Brillante S, Volpe M, Indrieri A (2024). Advances in MicroRNA Therapeutics: from Preclinical to Clinical Studies. Hum Gene Ther.

[19] Chen H, Xie G, Luo Q, Yang Y, Hu S (2023). Regulatory miRNAs, circRNAs and lncRNAs in cell cycle progression of breast cancer. Funct Integr Genomics.

[20] Khair HHA, Karagöz ID (2024). MiR-21-5p knockdown inhibits epithelial to mesenchymal transition in A549 lung adenocarcinoma cells by upregulating RhoB. Mol Biol Rep.

[21] Shen M, Zhou Z, Li BB, Lv M, Feng C, Chen S (2022). Investigation of miR-21-5p Key Target Genes and Pathways in Head and Neck Squamous Cell Carcinoma Based on TCGA Database and Bioinformatics Analysis. Technol Cancer Res Treat.

[22] Khair HHA, Karagoz ID (2024). MiR-21-5p knockdown inhibits epithelial to mesenchymal transition in A549 lung adenocarcinoma cells by upregulating RhoB. Mol Biol Rep.

[23] Bahrami N, Pirrafiee M, Azadi F, Azimnejad R, Fotook Kiaei SZ, Abbasi AJ (2024). Biomarkers for Oral Squamous Cell Carcinoma (miR-24, miR-200, and miR-34): Screening and Detection MicroRNA. Asian Pac J Cancer Prev.

[24] Arslan Bozdag L, Açik L, Ersoy HE, Bayir Ö, Korkmaz MH, Mollaoglu N (2024). PDCD4 and MIR-21 are promising biomarkers in the follow-up of OED in liquid biopsies. Oral Dis.

[25] Reis PP, Tomenson M, Cervigne NK, Machado J, Jurisica I, Pintilie M (2010). Programmed cell death 4 loss increases tumor cell invasion and is regulated by miR-21 in oral squamous cell carcinoma. Mol Cancer.

[26] Zhao W, Zhao J-J, Zhang L, Xu Q-F, Zhao Y-M, Shi X-Y (2015). Serum miR-21 level: a potential diagnostic and prognostic biomarker for non-small cell lung cancer. Int J Clin Exp Med.

[27] Huang Z, Kaller M, Hermeking H (2023). CRISPR/Cas9-mediated inactivation of miR-34a and miR-34b/c in HCT116 colorectal cancer cells: comprehensive characterization after exposure to 5-FU reveals EMT and autophagy as key processes regulated by miR-34. Cell Death Differ.

[28] Li Y, Jin L, Rong W, Meng F, Wang X, Wang S (2022). Expression and significance of miR-34 with PI3K, AKT and mTOR proteins in colorectal adenocarcinoma tissues. Cell Mol Biol (Noisy-le-grand).

[29] Rattan Negi R, Rana SV, Gupta V, Gupta R, Dhawan DK (2024). Evaluation of the Plasma Expression Levels of miR-21 and miR-145 as Potential Non-Invasive Biomarkers for Early Detection of Colorectal Cancer. Asian Pac J Cancer Prev.

[30] Ye G, Chen Y (2024). LncRNA FAM30A Predicts Adverse Prognosis and Regulates Cellular Processes in Colorectal Cancer via Modulating miR-21-3p. Turk J Gastroenterol.

[31] Zhang J, Guo J, He R, Li J, Du B, Zhang Y (2024). Analysis of the differential expression of serum miR-21-5p, miR-135-5p, and miR-155-5p by Bifidobacterium triplex viable capsules during the perioperative stage of colorectal cancer. Int J Colorectal Dis.

[32] Dai T, Qiu S, Gao X, Zhao C, Ge Z, Yang Y (2024). Circular RNA circWNK1 inhibits the progression of gastric cancer via regulating the miR-21-3p/SMAD7 axis. Cancer Sci.

[33] Zang YS, Zhong YF, Fang Z, Li B, An J (2012). MiR-155 inhibits the sensitivity of lung cancer cells to cisplatin via negative regulation of Apaf-1 expression. Cancer Gene Ther.

[34] Zanoaga O, Braicu C, Chiroi P, Andreea N, Hajjar NA, Margarit S (2021). The Role of miR-155 in Nutrition: Modulating Cancer-Associated Inflammation. Nutrients.

[35] Cheng C, Zhang Z, Wang J, Wang C, Liu T, Yang C (2024). CircPGM5 regulates Foxo3a phosphorylation via MiR-21-5p/MAPK10 axis to inhibit bladder cancer progression. Cell Signal.

[36] Qiao L, Dong C, Zhang J, Sun G (2023). The expression of Rab5 and its effect on invasion, migration and exosome secretion in triple negative breast cancer. Korean J Physiol Pharmacol.

[37] Ujfaludi Z, Fazekas F, Biro K, Olah-Nemeth O, Buzogany I, Sukosd F (2024). miR-21, miR-29a, and miR-106b: serum and tissue biomarkers with diagnostic potential in metastatic testicular cancer. Sci Rep.

[38] Wagner TM, Romero-Saavedra F, Laverde D, Johannessen M, Hubner J, Hegstad K (2023). Enterococcal Membrane Vesicles as Vaccine Candidates. Int J Mol Sci.

[39] Shaikh MAJ, Altamimi ASA, Afzal M, Gupta G, Singla N, Gilhotra R (2024). Unraveling the impact of miR-21 on apoptosis regulation in glioblastoma. Pathol Res Pract.

[40] Wang R, Chen Y, Kuang W, Jiang W, Zeng W, Chen Y (2024). Valproic acid regulates the miR-155/Jarid2 axis by affecting miR-155 promoter methylation in glioma. Acta Biochim Biophys Sin (Shanghai).

[41] Litak J, Grajkowska W, Bogucki J, Kowalczyk P, Petniak A, Podkowiński A (2022). PD-L1/miR-155 Interplay in Pediatric High-Grade Glioma. Brain Sci.

[42] Garavand J, Mohammadi MH, Jalali MT, Saki N (2024). Correlation of miR-155-5p, KRAS, and CREB Expression in Patients with Acute Myeloid Leukemia. Clin Lab.

[43] Liu T, Ma Y, Han S, Sun P (2024). Genome-wide investigation of lncRNAs revealed their tight association with gastric cancer. J Cancer Res Clin Oncol.

[44] Jackson BL, Grabowska A, Ratan HL (2014). MicroRNA in prostate cancer: functional importance and potential as circulating biomarkers. BMC Cancer.

[45] Arghiani N, Matin MM (2021). miR-21: A Key Small Molecule with Great Effects in Combination Cancer Therapy. Nucleic Acid Ther.

[46] Javanmard SH, Vaseghi G, Ghasemi A, Rafiee L, Ferns GA, Esfahani HN (2020). Therapeutic inhibition of microRNA-21 (miR-21) using locked-nucleic acid (LNA)-anti-miR and its effects on the biological behaviors of melanoma cancer cells in preclinical studies. Cancer Cell Int.

[47] Fu J, Imani S, Wu MY, Wu RC (2023). MicroRNA-34 Family in Cancers: Role, Mechanism, and Therapeutic Potential. Cancers (Basel).

[48] Adams BD, Parsons C, Slack FJ (2016). The tumor-suppressive and potential therapeutic functions of miR-34a in epithelial carcinomas. Expert Opin Ther Targets.

[49] Imani S, Wu RC, Fu J (2018). MicroRNA-34 family in breast cancer: from research to therapeutic potential. J Cancer.

[50] Bautista-Sánchez D, Arriaga-Canon C, Pedroza-Torres A, De La Rosa-Velázquez IA, González-Barrios R, Contreras-Espinosa L (2020). The Promising Role of miR-21 as a Cancer Biomarker and Its Importance in RNA-Based Therapeutics. Mol Ther Nucleic Acids.

[51] Kalkusova K, Taborska P, Stakheev D, Smrz D (2022). The Role of miR-155 in Antitumor Immunity. Cancers (Basel).

[52] Weng YS, Tseng HY, Chen YA, Shen PC, Al Haq AT, Chen LM (2019). MCT-1/miR-34a/IL-6/IL-6R signaling axis promotes EMT progression, cancer stemness and M2 macrophage polarization in triple-negative breast cancer. Mol Cancer.

[53] Liu C, Rokavec M, Huang Z, Hermeking H (2023). Curcumin activates a ROS/KEAP1/NRF2/miR-34a/b/c cascade to suppress colorectal cancer metastasis. Cell Death Differ.

[54] Imani S, Zhang X, Hosseinifard H, Fu S, Fu J (2017). The diagnostic role of microRNA-34a in breast cancer: a systematic review and meta-analysis. Oncotarget.

[55] Xiong S, Hu M, Li C, Zhou X, Chen H (2019). Role of miR-34 in gastric cancer: From bench to bedside (Review). Oncol Rep.

[56] Mattiske S, Suetani RJ, Neilsen PM, Callen DF (2012). The oncogenic role of miR-155 in breast cancer. Cancer Epidemiol Biomarkers Prev.

[57] Rong Shen R, Wang Y, Wang C-X, Yin M, Liu H-L, Chen J-P (2015). MiRNA-155 mediates TAM resistance by modulating SOCS6-STAT_3_ signalling pathway in breast cancer. Am J Transl Res.

[58] Bayraktar R, Van Roosbroeck K (2018). miR-155 in cancer drug resistance and as target for miRNA-based therapeutics. Cancer Metastasis Rev.

[59] He W, Yang G, Liu S, Maghsoudloo M, Shasaltaneh MD, Kaboli PJ (2021). Comparative mRNA/micro-RNA co-expression network drives melanomagenesis by promoting epithelial-mesenchymal transition and vasculogenic mimicry signaling. Transl Oncol.

[60] Fawzy MS, Ibrahiem AT, Bayomy NA, Makhdoom AK, Alanazi KS, Alanazi AM (2023). MicroRNA-155 and Disease-Related Immunohistochemical Parameters in Cutaneous Melanoma. Diagnostics (Basel).

[61] Gilles ME, Slack FJ (2018). Let-7 microRNA as a potential therapeutic target with implications for immunotherapy. Expert Opin Ther Targets.

[62] Xu F, Wang Y, Ling Y, Zhou C, Wang H, Teschendorff AE (2022). dbDEMC 3.0: functional exploration of differentially expressed miRNAs in cancers of human and model organisms. Genomics Proteomics Bioinformatics.

[63] Yang Z, Wu L, Wang A, Tang W, Zhao Y, Zhao H (2017). dbDEMC 2.0: updated database of differentially expressed miRNAs in human cancers. Nucleic Acids Res.

[64] Zhou Y, Ren H, Dai B, Li J, Shang L, Huang J (2018). Hepatocellular carcinoma-derived exosomal miRNA-21 contributes to tumor progression by converting hepatocyte stellate cells to cancer-associated fibroblasts. J Exp Clin Cancer Res.

[65] Xu P, Zhu Y, Sun B, Xiao Z (2016). Colorectal cancer characterization and therapeutic target prediction based on microRNA expression profile. Sci Rep.

[66] Zhang L, Liao Y, Tang L (2019). MicroRNA-34 family: a potential tumor suppressor and therapeutic candidate in cancer. J Exp Clin Cancer Res.

[67] Pan W, Chai B, Li L, Lu Z, Ma Z (2023). p53/MicroRNA-34 axis in cancer and beyond. Heliyon.

[68] Wang F, Wang J, Zhang H, Fu B, Zhang Y, Jia Q (2024). Diagnostic value of circulating miR-155 for breast cancer: a meta-analysis. Front Oncol.

[69] Ohlsson Teague EM, Van der Hoek KH, Van der Hoek MB, Perry N, Wagaarachchi P, Robertson SA (2009). MicroRNA-regulated pathways associated with endometriosis. Mol Endocrinol.

[70] Xu T, Zheng C, Wu Y, Chen Z, Miao HJC, Biology M (2024). MiR-34a ameliorates arterial blood flow in rats with lower limb arteriosclerosis obliterans via Sirt1 signaling pathway. Cell Mol Biol (Noisy-le-grand).

[71] Reddy S, Hu DQ, Zhao M, Ichimura S, Barnes EA, Cornfield DN (2024). MicroRNA-34a-Dependent Attenuation of Angiogenesis in Right Ventricular Failure. J Am Heart Assoc.

[72] Hidalgo-Sastre A, Lubeseder-Martellato C, Engleitner T, Steiger K, Zhong S, Desztics J (2020). Mir34a constrains pancreatic carcinogenesis. Sci Rep.

[73] Wang L, Xie Y, Chen W, Zhang Y, Zeng Y (2022). miR-34a Regulates Lipid Droplet Deposition in 3T3-L1 and C2C12 Cells by Targeting LEF1. Cells.

[74] Lopez CM, Yu PY, Zhang X, Yilmaz AS, London CA, Fenger JM (2018). MiR-34a regulates the invasive capacity of canine osteosarcoma cell lines. PLoS One.

[75] Rokavec M, Öner MG, Li H, Jackstadt R, Jiang L, Lodygin D (2014). IL-6R/STAT3/miR-34a feedback loop promotes EMT-mediated colorectal cancer invasion and metastasis. J Clin Invest.

[76] Nardone V, Barbarino M, Angrisani A, Correale P, Pastina P, Cappabianca S (2021). CDK4, CDK6/cyclin-D1 Complex Inhibition and Radiotherapy for Cancer Control: A Role for Autophagy. Int J Mol Sci.

[77] Li Y, Sun G, Wang L (2022). MiR-21 participates in LPS-induced myocardial injury by targeting Bcl-2 and CDK6. Inflamm Res.

[78] Li R, Li X, Ning S, Ye J, Han L, Kang C (2014). Identification of a core miRNA-pathway regulatory network in glioma by therapeutically targeting miR-181d, miR-21, miR-23b, β-Catenin, CBP, and STAT3. PLoS One.

[79] Ren J, Kuang TH, Chen J, Yang JW, Liu YX (2017). The diagnostic and prognostic values of microRNA-21 in patients with gastric cancer: a meta-analysis. Eur Rev Med Pharmacol Sci.

[80] Feng M-G, Liu C-F, Chen L, Feng W-B, Liu M, Hai H (2018). MiR-21 attenuates apoptosis-triggered by amyloid-β via modulating PDCD4/PI3K/AKT/GSK-3β pathway in SH-SY5Y cells. Biomed Pharmacother.

[81] Qin X, Yan L, Zhao X, Li C, Fu Y (2012). microRNA-21 overexpression contributes to cell proliferation by targeting PTEN in endometrioid endometrial cancer. Oncol Lett.

[82] Jafarzadeh A, Naseri A, Shojaie L, Nemati M, Jafarzadeh S, Bannazadeh Baghi H (2021). MicroRNA-155 and antiviral immune responses. Int Immunopharmacol.

[83] Zingale VD, Gugliandolo A, Mazzon E (2021). MiR-155: An Important Regulator of Neuroinflammation. Int J Mol Sci.

[84] Witten LW, Cheng CJ, Slack FJ (2019). miR-155 drives oncogenesis by promoting and cooperating with mutations in the c-Kit oncogene. Oncogene.

[85] Schulte LN, Westermann AJ, Vogel J (2013). Differential activation and functional specialization of miR-146 and miR-155 in innate immune sensing. Nucleic Acids Res.

[86] Zanoaga O, Braicu C, Chiroi P, Andreea N, Hajjar NA, Mărgărit S (2021). The Role of miR-155 in Nutrition: Modulating Cancer-Associated Inflammation. Nutrients.

[87] Menon A, Abd-Aziz N, Khalid K, Poh CL, Naidu R (2022). miRNA: A Promising Therapeutic Target in Cancer. Int J Mol Sci.

[88] Reda El Sayed S, Cristante J, Guyon L, Denis J, Chabre O, Cherradi N (2021). MicroRNA Therapeutics in Cancer: Current Advances and Challenges. Cancers (Basel).

[89] Ho PTB, Clark IM, Le LTT (2022). MicroRNA-Based Diagnosis and Therapy. Int J Mol Sci.

[90] Chandrashekar DS, Karthikeyan SK, Korla PK, Patel H, Shovon AR, Athar M (2022). UALCAN: An update to the integrated cancer data analysis platform. Neoplasia.

[91] Hong DS, Kang YK, Borad M, Sachdev J, Ejadi S, Lim HY (2020). Phase 1 study of MRX34, a liposomal miR-34a mimic, in patients with advanced solid tumours. Br J Cancer.

[92] Lucibello G, Mograbi B, Milano G, Hofman P, Brest P (2021). PD-L1 regulation revisited: impact on immunotherapeutic strategies. Trends Mol Med.

[93] Gunawan RR, Astuti I, Danarto HR (2023). miRNA-21 as High Potential Prostate Cancer Biomarker in Prostate Cancer Patients in Indonesia. Asian Pac J Cancer Prev.

[94] Ding Y, Wu W, Ma Z, Shao X, Zhang M, Wang Z (2021). Potential value of MicroRNA-21 as a biomarker for predicting the prognosis of patients with breast cancer: A protocol for meta-analysis and bioinformatics analysis. Medicine (Baltimore).

[95] Li W, Huang X, Bi D (2022). miRNA-21 plays an important role in necrotizing enterocolitis. Arch Med Sci.

[96] Beg MS, Brenner AJ, Sachdev J, Borad M, Kang YK, Stoudemire J (2017). Phase I study of MRX34, a liposomal miR-34a mimic, administered twice weekly in patients with advanced solid tumors. Invest New Drugs.

[97] Winkle M, El-Daly SM, Fabbri M, Calin GA (2021). Noncoding RNA therapeutics - challenges and potential solutions. Nat Rev Drug Discov.

[98] Momin MY, Gaddam RR, Kravitz M, Gupta A, Vikram A (2021). The Challenges and Opportunities in the Development of MicroRNA Therapeutics: A Multidisciplinary Viewpoint. Cells.

[99] Nappi F (2024). Non-Coding RNA-Targeted Therapy: A State-of-the-Art Review. Int J Mol Sci.

[100] Sweef O, Zaabout E, Bakheet A, Halawa M, Gad I, Akela M (2023). Unraveling Therapeutic Opportunities and the Diagnostic Potential of microRNAs for Human Lung Cancer. Pharmaceutics.

[101] Shen M, Chen T, Li X, Zhao S, Zhang X, Zheng L (2024). The role of miR-155 in urologic malignancies. Biomed Pharmacother.

[102] Dasgupta I, Chatterjee A (2021). Recent Advances in miRNA Delivery Systems. Methods Protoc.

[103] Gareev I, Beylerli O, Tamrazov R, Ilyasova T, Shumadalova A, Du W (2023). Methods of miRNA delivery and possibilities of their application in neuro-oncology. Noncoding RNA Res.

[104] Zhang Y, Wang Z, Gemeinhart RA (2013). Progress in microRNA delivery. J Control Release.

[105] Wen KX, Miliç J, El-Khodor B, Dhana K, Nano J, Pulido T (2016). The Role of DNA Methylation and Histone Modifications in Neurodegenerative Diseases: A Systematic Review. PLoS One.

[106] Naso MF, Tomkowicz B, Perry WL 3rd, Strohl WR (2017). Adeno-Associated Virus (AAV) as a Vector for Gene Therapy. BioDrugs.

[107] Seo HA, Moeng S, Sim S, Kuh HJ, Choi SY, Park JK (2019). MicroRNA-Based Combinatorial Cancer Therapy: Effects of MicroRNAs on the Efficacy of Anti-Cancer Therapies. Cells.

[108] He B, Zhao Z, Cai Q, Zhang Y, Zhang P, Shi S (2020). miRNA-based biomarkers, therapies, and resistance in Cancer. Int J Biol Sci.

[109] Alard E, Butnariu AB, Grillo M, Kirkham C, Zinovkin DA, Newnham L (2020). Advances in Anti-Cancer Immunotherapy: Car-T Cell, Checkpoint Inhibitors, Dendritic Cell Vaccines, and Oncolytic Viruses, and Emerging Cellular and Molecular Targets. Cancers (Basel).

[110] Hussen BM, Rasul MF, Abdullah SR, Hidayat HJ, Faraj GSH, Ali FA (2023). Targeting miRNA by CRISPR/Cas in cancer: advantages and challenges. Mil Med Res.

[111] Pagoni M, Cava C, Sideris DC, Avgeris M, Zoumpourlis V, Michalopoulos I (2023). miRNA-Based Technologies in Cancer Therapy. J Pers Med.

[112] Fujii E, Kato A, Suzuki M (2020). Patient-derived xenograft (PDX) models: characteristics and points to consider for the process of establishment. J Toxicol Pathol.

[113] Mc Cormack BA, González-Cantó E, Agababyan C, Espinoza-Sánchez NA, Tomás-Pérez S, Llueca A (2021). miRNAs in the Era of Personalized Medicine: From Biomarkers to Therapeutics. Int J Mol Sci.

[114] Yang M, Zhang Y, Li M, Liu X, Darvishi M (2023). The various role of microRNAs in breast cancer angiogenesis, with a special focus on novel miRNA-based delivery strategies. Cancer Cell Int.

[115] So JBY, Kapoor R, Zhu F, Koh C, Zhou L, Zou R (2021). Development and validation of a serum microRNA biomarker panel for detecting gastric cancer in a high-risk population. Gut.

[116] Daniel Thomas S, Vijayakumar K, John L, Krishnan D, Rehman N, Revikumar A (2024). Machine Learning Strategies in MicroRNA Research: Bridging Genome to Phenome. Omics.

[117] Lu H, Zhang J, Cao Y, Wu S, Wei Y, Yin R (2024). Advances in applications of artificial intelligence algorithms for cancer-related miRNA research. Zhejiang Da Xue Xue Bao Yi Xue Ban.

[118] Sempere LF, Azmi AS, Moore A (2021). microRNA-based diagnostic and therapeutic applications in cancer medicine. Wiley Interdiscip Rev RNA.

[119] Vlachos IS, Zagganas K, Paraskevopoulou MD, Georgakilas G, Karagkouni D, Vergoulis T (2015). DIANA-miRPath v3. 0: deciphering microRNA function with experimental support. Nucleic Acids Res.

[120] Chen J, Bardes EE, Aronow BJ, Jegga AGJNar (2009). ToppGene Suite for gene list enrichment analysis and candidate gene prioritization. Nucleic Acids Res.

[121] Dweep H, Gretz NJNm (2015). miRWalk2. 0: a comprehensive atlas of microRNA-target interactions. Nat Methods.

